# Short-Term Hemodynamic Effects of Modern Wheat Products Substitution in Diet with Ancient Wheat Products: A Cross-Over, Randomized Clinical Trial

**DOI:** 10.3390/nu10111666

**Published:** 2018-11-04

**Authors:** Arrigo F. G. Cicero, Federica Fogacci, Maddalena Veronesi, Elisa Grandi, Giovanni Dinelli, Silvana Hrelia, Claudio Borghi

**Affiliations:** 1 Medical and Surgical Sciences Department, University of Bologna, Bologna 40138, Italy; federicafogacci@gmail.com (F.F.); maddalena.veronesi@unibo.it (M.V.); elisa.grandi@unibo.it (E.G.); claudio.borghi@unibo.it (C.B.); 2 Department of Agricultural and Food Sciences (DISTAL), University of Bologna, Bologna 40138, Italy; giovanni.dinelli@unibo.it; 3 Department for Life Quality Studies, University of Bologna, Rimini 47921, Italy; silvana.hrelia@unibo.it

**Keywords:** bioactive peptide, blood pressure, endothelial reactivity, randomized clinical trial, ancient wheat, non-specific lipid-transfer protein type 2

## Abstract

Recent evidence suggests that bioactive compounds isolated from cereals and legumes could exert some metabolic and vascular effects in humans. Due to the recent identification of a non-specific lipid transfer protein (nsLTP2) in wheat with antioxidant and anti-inflammatory activity, we aimed to comparatively test the hemodynamic and metabolic effects of ancient wheat foodstuffs (made of organic KAMUT^®^ khorasan wheat) or modern wheat ones, made of a mixture of organic modern commercial durum (*T. durum*) varieties and soft wheat (*T. aestivum*), with different nsLTP2 content. Thus, we carried out a randomized, cross-over clinical trial on 63 non-diabetic healthy volunteers (aged 40–70 years) with systolic blood pressure (SBP) 130–139 mmHg and/or diastolic blood pressure (DBP) 85–90 mmHg (pre-hypertensive/borderline high pressure subjects). Each treatment period lasted four weeks. After ancient wheat foodstuffs intake, subjects experienced a significant improvement in triglycerides (−9.8% vs. baseline and −14.5% versus modern wheat), fasting plasma glucose (−4.3% versus baseline and −31.6% versus modern wheat), diurnal SBP (−3.1% vs. baseline and –30.2% vs. modern wheat) and nocturnal SBP (−3.2% vs. baseline and −36.8% vs. modern wheat), and pulse volume change (+4.2% vs. baseline and +2.3% vs. modern wheat) (*p* < 0.05 vs. baseline and versus modern wheat foodstuffs intake). Therefore, our findings show that substituting modern wheat products in diet with ancient wheat ones, might exert a mild improvement in 24-h SBP and endothelial reactivity in pre-hypertensive healthy subjects.

## 1. Introduction

The international dietary guidelines emphasize cereals’ and legumes’ health-promoting properties by placing them in the base of the nutritional food pyramid [1,2]. This concept has been further validated by the observed correlation between a lower risk and occurrence of cardiovascular disease and the adherence to dietary patterns, like the Mediterranean diet [3], in which cereal grains, legumes and derived products represent a staple food. A relevant part of the positive effect of cereals on human health and blood pressure (BP) control has been recently proposed for protein derived bioactive peptides, which may already be present in foods as natural components or derive from hydrolysis by chemical or enzymatic treatments (such as digestion, hydrolysis or fermentation) [4]. One of the most studied vegetable bioactive peptides is lunasin, particularly concentrated in legumes [5]. In the last decade, the presence of lunasin has also been reported in cereals [6], though we have just demonstrated by chemical and molecular analyses that lunasin is absent in wheat kernels [7]. In fact, the content of bioactive peptides is largely different in different cereals and cereal-derived foods. The analysis of different wheat extracts purified by chromatographic techniques allowed the identification of non-specific lipid-transfer protein type 2 (nsLTP2) from *Triticum aestivum* [8]. This peptide is chemically thermo- and protease-resistant. Moreover, in human umbilical vein endothelial cells (HUVEC) nsLTP2 has been demonstrated to exert antioxidant activity and potential cytoprotective effects following oxidative or inflammatory stimulations [8,9]. More recently, a proof of concept study demonstrated the ability of nsLTP2 to downregulate the expression of the main cell adhesion molecules induced by a pro-inflammatory cytokine and upregulate heme oxigenase-1 in HUVEC cells [10]. This study suggests that nsLTP2 might represent an important bioactive molecule to protect the vascular system against different stressors [10]. Despite the preclinical evidence of a possible positive effect of nsLTP2 from wheat on the vascular system, it needs to be confirmed in randomized clinical trials on humans where a large number of confounding factors are controlled.

In this context, the aim of our study was to test the short-term hemodynamic and metabolic effect of two kinds of commercial wheat products made by wheat naturally containing high or low amounts of bioactive peptide nsLTP2 in pre-hypertensive subjects.

## 2. Materials and Methods

### 2.1. Study Design

This was a double-blind, randomized, feeding-controlled, cross-over clinical trial (Registration number: NCT02197910), carried out in a group of pre-hypertensive adult volunteers (130 mmHg < systolic BP (SBP) ≤139 mmHg and 85 mmHg < diastolic BP (DBP) ≤89 mmHg) at moderately increased estimated cardiovascular risk (ESH charts) [11], consecutively enrolled in the ambulatory service of cardiovascular disease prevention of the Medical and Surgical Sciences Department, University of Bologna.

Patients with known primitive or white coat hypertension, obesity (body mass index (BMI) >25 kg/m^2^), diabetes mellitus, personal history of atherosclerosis-related cardiovascular diseases (coronary artery disease, cerebrovascular disease, ultrasound diagnosed carotid atherosclerosis), known active thyroid disorders, renal failure or chronic liver disease and subjects consuming drugs, botanical extracts or other dietary supplements potentially affecting BP were excluded from the study as well as subjects with gastrointestinal disease (e.g., celiac disease) that could cause problems in the absorption of bioactive peptides.

**Figure 1:**
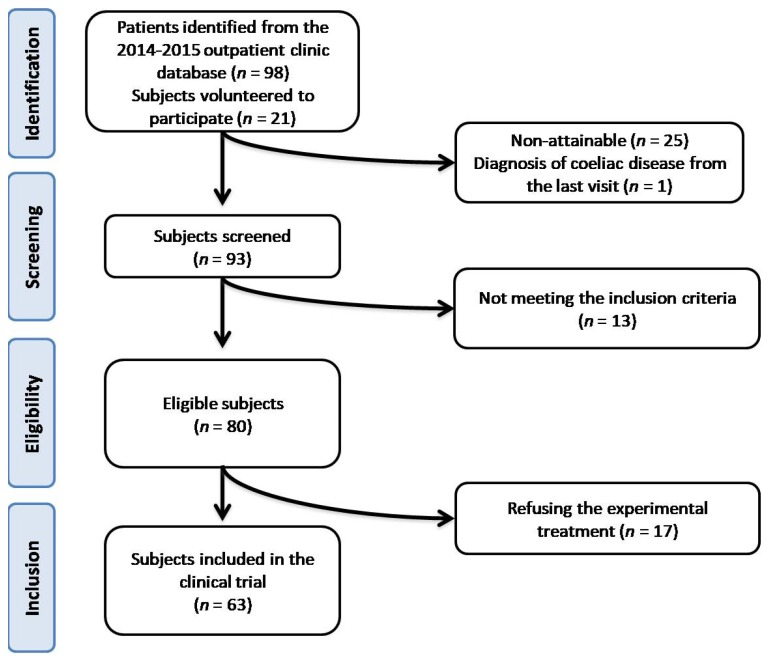
Flowchart resuming the selection criteria for the subjects enrolled in the trial.

After retrospectively evaluated 3212 patients from the 2014–2015 outpatient clinic database, we indirectly identified 98 potentially recruitable subjects. In addition, 21 subjects from the hospital personnel were offered voluntarily to participate. Firstly, we selected 93 eligible subjects: among them, 13 patients were excluded at the screening visit because the inclusion criteria was not met and 17 subjects refused to give their informed consent to the treatment. At the end, 63 subjects were selected and enrolled (Figure 1).

**Figure 2:**
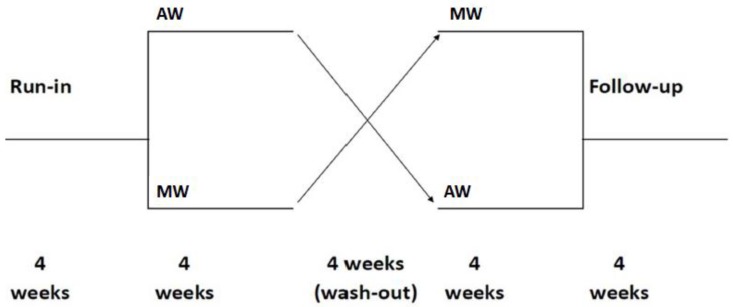
Flowchart of the trial. AW: ancient wheat foodstuffs, MW: modern wheat foodstuffs.

Participants were adhering to a standardized diet for four weeks before being randomly assigned to complete one of two treatment sequences by consuming a prescribed quantity of tested products for a four-week period, followed by a four-week washout before the cross-over to the second treatment. Finally, they were asked to return for a follow-up visit at four weeks, in order to evaluate the reversibility of the eventual bioactive peptide effect after suspension (Figure 2).

During the period of diet standardization, patients were given standard behavioral and qualitative dietary suggestion to correct their lifestyle habits. In particular, they were instructed to follow the general indications of a Mediterranean diet (avoiding an excessive intake of dairy and red-meat-derived products), to increase their daily intake of vegetables and not to add salt to fresh and cooked food. They were also recommended to increase their physical activity by walking briskly or cycling 3–5 times per week for at least 20–30 min each time. Thereafter, subjects were asked to maintain constant their habits for the entire duration of the trial, in order to reduce the studied parameters’ variability related to any dietary excesses. In particular, it was asked to the enrolled volunteers to maintain a constant intake of fruits, vegetables, olive oil and wine, in order to reduce the variability in the dietary content of fibers and polyphenols. Nutrient intake was estimated from the 4 days records before the randomization and towards the end of each intervention period. The nutritional evaluation was performed by an expert nutritionist biologist with Software MètaDieta^®^ (Meteda S.r.l., San Benedetto del Tronto, Italy) using Italian Food Composition databases. Compliance with the dietary instructions was further verified by the evaluation of 24 h urinary sodium, as biomarker of salt intake.

Randomization was done by an independent statistician using a specific software. The statistician prepared envelopes containing randomization codes to assign in a progressive way to enroll subjects. All participants, study staffs and data analysis were blinded to the group assignment. A copy of the codes was provided only to the person responsible for performing the statistical analysis.

Throughout the study, we instructed patients to take the first quantity of pasta on the day after they received it in a blinded box. All unused foodstuffs were retrieved for inventory. Product compliance was assessed by counting the number of packaged foodstuffs returned at the time of specified clinic visits. The acceptability of the tested products was assessed by a visual analogue scale.

The present study was fully conducted in accordance with the Declaration of Helsinki, its protocol was approved by the Ethical Committee of the University of Bologna (Code: BACCHUS_Unibo, Bologna, Italy) and a written informed consent was obtained from all patients before they were included in the trial. The trial was registered on www.clinicaltrial.gov (Code: NCT02197910) as well.

### 2.2. Wheat Products

The intervention was carried out using wheat products containing different quantities of nsLTP2 (kindly provided by Kamut Enterprises of Europe (KEE), Oudenaarde, Belgium), which appeared and tasted the same. During the treatment periods, volunteers consumed pasta, bread, and crisp toasts naturally containing a low or a high dose of nsLTP2, substituting the amount of usually consumed foodstuff with the ones provided for the study. The experimental ancient wheat utilized in the present study was organic KAMUT^®^ khorasan wheat (*Triticum turgidum* subsp. *turanicum*), while a mix of organic modern commercial durum (*T. durum*) varieties and soft wheat (*T. aestivum*) were used as nsLTP2 low dose wheat. KAMUT^®^ khorasan (Oudenaarde, Belgium) is characterized by a particularly high content in nsLTP2 compared to the modern wheat.

KAMUT^®^ is a registered trademark of Kamut International, Ltd. and Kamut Enterprises of Europe and guarantees the wheat is pure ancient khorasan wheat and is organically grown and processed.

According to the procedure reported by Bosi et al. (2018), [10] the nsLTP2 content was quantified by high performance liquid chromatography-ultraviolet (HLPC-UV)/nano-liquid chromatography-nano-electrospray-ionisation quadrupole time-of-flight (ESI-QTOF) mass spectrometry, in both foodstuffs composed by modern wheat and KAMUT^®^ khorasan wheat. Assuming a mean daily consumption of 100 g of pasta, 100 g of matzo bread and 40 g of crispy, volunteers under modern wheat and KAMUT^®^ khorasan wheat diet assumed respectively 20.5 and 41.0 mg peptide per day.

The total phenolic compounds contained in the tested products was also estimated based on their content in the flours. Total phenolic compounds were extracted as previously described by Dinelli et al. [12]. One gram of whole grain flours was extracted with cold 80% ethanol (4 °C) to dissolve the free soluble compounds, followed by acid and alkaline hydrolyses to release the bound forms. The free and bound extracts were pooled together, evaporated to dryness, and reconstituted to a final concentration of 10 mg/mL in pure methanol. The extracts were filtered through a 0.22 mm filter and stored at −20 °C until analysis.

The 2,2-diphenyl-1-picrylhydrazyl (DPPH) assay was carried out according to the procedure described by Floegel et al. [13]. Briefly, a solution of 1 mM DPPH in 80% *v*/*v* methanol was prepared. Absorbance of the solution was adjusted to 0.65 ± 0.02 arbitrary units (AU) at 517 nm using fresh 80% *v*/*v* methanol. Subsequently, 50 mL of free and bound extract were mixed with 2.95 mL of DPPH solution and incubated for 30 min in the dark at room temperature. The absorbance at 517 nm was monitored for each sample, along with control against a blank of pure methanol. A calibration curve of Trolox (0–500 mg/L) was performed as a function of the percentage of DPPH radical scavenging activity and the final results expressed as micromoles of Trolox equivalents (TE) per gram of whole wheat flour (mmol TE/g). The Fluorescence Recovery After Photobleaching (FRAP) test was carried out according to Benzie and Strain [14] with some modifications. The FRAP working solution (WS) was prepared freshly as a mixture of 300 mM acetate buffer pH 3.6 (containing 3.1 g of sodium acetate trihydrate and 16 mL glacial acetic acid), 10 mM TPTZ (in 40 mmol/L HCl), and 20 mM ferric chloride (10:1:1, *v*:*v*:*v*). Eighty microliters of diluted (1:1, *v*/*v*) free and bound phenolic extracts were mixed with 2.4 mL of WS and the absorbance was measured at 593 nm after 1 h in darkness. Absorbance values were compared with those of ferrous sulphate (FeSO_4_·7H_2_O) (0–1000 mmol/L) and results expressed as mmol Fe^2+^ per 100 g of flour. Each measurement was performed in triplicate.

All transformation preparation procedures were identical for both ancient and modern wheat products. Other cereals were excluded from the diet and replaced by either the ancient or modern wheat products during the intervention phases. The food products were packaged with no labels attached to the packages.

### 2.3. Assessments

#### 2.3.1. Clinical Data and Anthropometric Measurements

Patients were evaluated by the execution of blood and vascular tests at enrolment and at each control. Each visit included standardized questionnaires updating of personal anamnesis (with specific attention to dietary habit assessment, smoking status, and pharmacological treatments) and a physical examination with anthropometric data collection (height, weight, waist, and hip circumference). Height and body weight were measured by standard procedures to the nearest 0.1 cm and 0.1 kg, respectively, with subjects standing erect with eyes directed straight ahead, wearing light clothes, and with bare feet. Waist circumference (WC) was measured at the end of a normal expiration, in a horizontal plane at the midpoint between the inferior margin of the last rib and the superior iliac crest. Instrumental variables investigated were BP, 24 h ambulatory blood pressure monitoring (ABPM), endothelial function, arterial stiffness, and related parameters.

#### 2.3.2. Blood Pressure Measurement

BP was measured from the right upper arm, in the sitting position and after a 10-min rest in a quiet room. Measurements (obtained early in the morning) were performed using a standard mercury sphygmomanometer (Erkameter 3000, ERKA, Bad Tolz, Germany; Korotkoff I and V) and cuffs of appropriate size and snug fit. To implement detection’s accuracy, the average between three successive BP readings (each one obtained at a 1 min interval) was considered as study-variable. Mean pulse pressure (PP) was calculated as the difference between systolic (SBP) and diastolic blood pressure (DBP) (PP = SBP − DBP). Mean arterial pressure (MAP) was obtained by adding one-third of PP to DBP (MAP = ⅓PP + DBP) [11].

#### 2.3.3. 24 h Ambulatory Blood Pressure Monitoring (ABPM)

ABPM was performed for 24 h, using a noninvasive automatic monitor (model 90207; Space Labs, Redmond, WA, USA). For ABPM, patients were given instruction about how to normally act and work between 06:00 and 22:00 and rest or sleep between 22:00 and 06:00, without taking any drug or xanthine rich beverages (coffee, tea, and energy drinks), which could potentially affect their BP level.

Readings were automatically obtained at 15 min intervals throughout a 24 h study period. Separate averages were taken into consideration for 24 h daytime (06:00–22:00) and nighttime (22:00–06:00) values. The accuracy of the automatic BP readings were checked twice for each ABPM and compared to the manual readings, which were taken using a standard mercury sphygmomanometer. BP was measured by the same operator, from the right upper arm, with the patient in a sitting position, and after a 5-min rest period before the beginning of the ABPM. Three readings were obtained and averaged. The accuracy test was repeated after the end of each 24 h ABPM. Patients with more than 5 mmHg difference in SBP between the manual and automatic readings were excluded from further analysis. All sampled records were blindly decoded by a trained physician [11].

#### 2.3.4. Pulse Wave Velocity and Related Measures

Arterial stiffness parameters and endothelial function were assessed using the Vicorder^®^ apparatus (Skidmore Medical Ltd, Bristol, UK), a validated cuff-based device that estimates central BP using a brachial-to-aortic transfer function. Pulse wave velocity (PWV) consists in the measurement of the pulse wave transmission through the arteries and it is considered a reliable and early marker of arterial stiffness as well as a strong predictor of cardiovascular risk [15]. Theoretical basis of PWV are explained with the Moens–Kortewe equation [16], while in clinical practice PWV is calculated as the length between two measurement sites divided by the time the pulse wave takes to cover that distance (m/s) [17]. Augmentation Index (AIx) is obtained through the BP waveform analysis. It represents, as well as PWV, a measure of wave reflection and arterial stiffness and a marker of cardiovascular risk [18]. It is calculated as the ratio of the pressure increment caused by the reflected wave (augmented pressure) to the pulse pressure [19]. Pulse Wave Analysis, from which AIx is obtained, is recorded simply with a brachial cuff placed at the right arm of the subjects: the Vicorder^®^ apparatus registers the radial pressure and with a specific algorithm derives the central BP curve. PWV is calculated with a simultaneous measurement of carotid and femoral BP. A small neck pad containing a photoplethysmographic sensor is placed around the neck and a normal cuff is positioned around the thigh of the patient. The distance between the suprasternal notch and the thigh cuff is measured with a measuring tape. This length represents the distance covered by the pulse wave in its carotid–femoral path and it is used by the Vicorder^®^ apparatus to establish the PWV value [20,21]. The Vicorder^®^ apparatus guarantees a very good intra- and interoperator reliability [22].

#### 2.3.5. Endothelial Function

Endothelial function was evaluated though the Endocheck^®^ (BC Biomedical Laboratories Ltd, Vancouver, BC, Canada), a method embedded within the Vicorder^®^ device, which is supposed to record brachial pulse volume (PV) waveforms, at baseline and during reactive hyperemia. Reactive hyperemia usually is provoked through PV displacement, obtained by inflating a cuff positioned distally around the forearm. After a 10 min rest, brachial BP is evaluated and PV waveforms are recorded at the baseline for 10 s. Then, the cuff is inflated to 200 mmHg for 5 min and PV waveforms are recorded for 3 min after cuff released. PV displacement is, then, calculated as a percent change in the PV waveform area, comparing waveforms before and during hyperemia through the equation:

PV displacement = PV_2_/PV_1_ (1)

where PV_1_ represents PV at the baseline and PV_2_ represents PV during hyperemia [23].

#### 2.3.6. Laboratory Data

All measurements were centrally performed in the laboratory of our department. The biochemical analysis were carried out on venous blood and all subjects had fasted for at least 12 h at the time of sampling. Plasma used was obtained by addition of Disodium ethylenediaminetetraacetate dehydrate (Na_2_EDTA) (1 mg/mL) and centrifuged at 3000× *g* for 15 min at 25 °C. Trained personnel performed laboratory analysis according to standardized methods [24], immediately after centrifugation, evaluating total cholesterol (TC), high-density lipoprotein cholesterol (HDL-C), triglycerides (TG), low-density lipoprotein cholesterol (LDL-C), apolipoprotein B (apoB), apolipoprotein A1 (apoA1), fasting plasma glucose (FPG), creatinine, liver transaminases and creatinine phosphokinase (CPK). At the end of the first visit, participants were given two three-liter plastic bottles and standardized instructions on how to collect urine. They were asked to first empty their bladder, discard the urine and write down the time (start of the collection). The end of the collection was determined by the last urine void in the study center at the second visit or, if not possible, by the time of the last void. Participants were asked whether any collection of urine was lost or forgotten. Then the sodium content was measured with standardized methods.

#### 2.3.7. Statistical Analyses

Sample size was calculated for the primary endpoint of the study (improvement in endothelial reactivity). Considering a Type I error of 0.05 and a power of 0.80 and expecting a minimum pulse volume change improvement of 3.3% with a SD of 5.5%, and considering a drop-out rate of 10%, we calculated to enroll at least 61 patients (to be treated with both tested foodstuffs in the context of a crossover trial). Data were analyzed using intention to treat by means of the Statistical Package for Social Science (SPSS) version 21.0 (IBM Corporation, Armonk, NY, USA) for Windows. Normally distributed baseline characteristics of the population were described by using independent T-test and χ^2^ test (Fisher corrected for categorical variables). Two-way analysis of variance for crossover design were used in order to assess the treatment effect during the use of low or high nsLPT2. Given their not normal distribution, total polyphenol content and antioxidant activities of the tested ancient and modern wheats were compared using non parametric analysis of variance (Kruskal‒Wallis test) followed by Mann‒Whitney-U. All data were expressed as mean ± standard deviation (SD). To verify the basic assumptions of crossover design, besides the evaluation of period effect, the presence of a carryover effect was excluded. A *P*-value of 0.05 was considered significant for all tests.

## 3. Results

The nutritional composition of the tested foodstuffs is reported in Table 1.

The total polyphenol content and antioxidant activities (DPPH and FRAP) of the tested modern and ancient wheat are reported in Table 2. No statistical differences were observed in the total polyphenol content. Regarding the antioxidant properties, the DPPH test evidenced a statistical difference: modern wheat exhibited a higher DPPH activity with respect to ancient wheat. The opposite was observed for the antioxidant FRAP activity, which was approximately 50% higher in ancient wheat whole semolina. Considering the complementary role of DPPH and FRAP activities, we can affirm that the two types of flour have similar antioxidant activity.

All 63 enrolled subjects (men: 30; women: 33) successfully completed the study. The compliance with the treatment was almost total during both modern and ancient foodstuffs periods and no adverse effects were reported during the trial.

Patients’ characteristics at the screening visit and after diet standardization are reported in Table 3. At baseline, subjects assigned to the different treatment sequences were well matched for all of the considered variables, including sex and age.

Enrolled subjects maintained similar dietary habits from the randomization until the end of the study, without significant changes in total energy, salt intake and coffee and alcohol consumption (Table 4).

During the trial, they did not experience any changed either in office BP, urine Na^++^ and anthropometric and hematochemical parameters, except for FPG and TG, which significantly improved (*P* < 0.05) after ancient wheat foodstuffs intake, versus baseline (−4.3% and −9.8%, respectively) and versus modern wheat foodstuffs assumption (−31.6% and −14.5%, respectively) (Table 5).

Moreover, during the ancient wheat food stuffs treatment subjects experienced a significant improvement in diurnal SBP (−3.1% vs. baseline and –30.2% vs. low nsLTP2), nocturnal SBP (−3.2% vs. baseline and −36.8% vs. modern wheat foodstuffs intake), 24h-SBP (−2.3% vs. baseline), %SBP> normal (−4.6% vs. baseline and −12.5% vs. modern wheat foodstuffs intake), % diurnal SBP> normal (−4.9% vs. baseline and −34.6% vs. low nsLTP2), and % night SBP> normal (−4.5% vs. baseline and −36.8% vs. modern wheat foodstuffs intake) (Table 6) and pulse volume change, as a marker of endothelial reactivity (+4.2% vs. baseline and +2.3% vs. modern wheat foodstuffs intake) (*P* < 0.05 always). However, pulse wave velocity and augmentation index changed neither during the high nor during the low modern wheat foodstuffs intake sequence (Table 7).

All the parameters reversed to the baseline levels after both the first and the second wash-out period.

## 4. Discussion

There is a growing interest in studying foods and food components potentially active in reducing cardiovascular disease risk [24].

In the recent years, several epidemiological studies have demonstrated that a regular consumption of whole grains positively affects the human health, reducing the risk of developing type 2 diabetes and managing obesity [25]. In particular, the ancient KAMUT^®^ khorasan wheat has been lately linked to a lower cardiovascular mortality rate in elderly [26].

In our randomized, cross-over, double blind clinical trial carried out on a sample of overall healthy subjects with suboptimal BP control, after ancient wheat foodstuffs intake, diurnal and nocturnal SBP and pulse volume change (as a marker of endothelial reactivity) significantly improved versus baseline and versus modern wheat foodstuff consumption. Even if this change may seem negligible, a recent meta-analysis of prospective studies concluded that a 1% improvement in endothelial reactivity is associated with about 12% reduction in risk of cardiovascular disease [27]. Consequently, our findings should be taken into account as of potential interest. PWV and AIx did not change during both wheat products intake, probably because of the relatively short-term exposition to the experimental products. On the other hand, plasma fasting TG and glucose significantly improved only after ancient wheat food stuffs intake versus baseline and versus modern wheat foodstuffs intake.

These data are partially in agreement with what reported from previous studies. In particular, in a previous small trial [28] no effect on BP was observed, however, that study enrolled healthy subjects while we selected prehypertensive volunteers. In the same trial there was a significant reduction of metabolic risk factors such as LDL-C (−7.8%) and FPG during ancient wheat intervention. Similarly, redox status was significantly improved only after the ancient wheat intervention phase, as measured by a reduction in both thiobarbituric acid reactive substances (−0.17 nmol/mL; −21.5%;) and carbonyl levels (−0.16 nmol/mL; −17.6%). Thus, some of the observed effects may have been due to other components of ancient wheat rather than bioactive peptides such as antioxidant flavonoids [29]. In particular, ancient grains are often characterized by a higher protein content and sometimes by a higher phenolic content, although high variability has been reported in the different genotypes [12]. In our study, however, we compared foodstuffs prepared with flours having a similar total polyphenol content and similar antioxidant properties. Thus, even if nsLTP2 is not the only bioactive peptide contained in ancient wheat, we suppose that it could be partially responsible for the observed vascular effects because it is significantly more concentrated in ancient than in modern wheat, being thermoresistant and protease-resistant, and characterized by a specific antioxidant and anti-inflammatory action [8]. Of course, this could not completely exclude the fact that other derived peptides could contribute to the final observed effect.

Our trial has some limitations. First, the duration of the experimental treatments was too short to observe more relevant changes in vascular parameters, namely the ones related to arterial stiffness. At the same time, the short run-in period could have caused an overlap between the effect of dietary and life-style improvement on metabolic and hemodynamic parameters and the one of the tested products. However, the same suggestions have been furnished to both treatment groups and during both dietary phases, so that this limitation should not be so relevant. Then, we did not measured advanced laboratory parameters such as chemokines, endothelial adhesion molecules or inflammatory markers. However, Sofi et al. [30] already observed that circulating levels of key pro-inflammatory cytokines (interleukin-6, interleukin-12, tumor necrosis factor-α and vascular endothelial growth factor) were significantly reduced after the consumption of ancient wheat foodstuffs. Therefore, we did not analyze the levels of nsLTP2 or metabolites in blood. Actually, this analysis could be very difficult due to a lack of standardized and reliable validated protocols helping in the evaluation of this peptide in biological samples. Besides, this is an aim of our future researches. Finally, we are not able to fully exclude that a part of the positive effect of ancient wheat foodstuffs intake on vascular health is mediated by antioxidant components or direct actions on glucose metabolism and insulin-resistance (as already demonstrated in coronary heart disease [31] and type 2 diabetes patients [32]) and not only by a direct effect of bioactive peptides on endothelium. Although, as for other foods (namely wine, olive oil, etc.), a sum of bioactive compounds is likely to be responsible of the observed effect of ancient wheat foodstuffs on human health, in our trial the main difference between the tested wheat products was the nsLTP2 concentration, besides a small difference in selenium content. So that, notwithstanding we cannot exclude the contribution of other bioactive compounds, we can argue that nsLTP2 could play an important role in the observed results. This hypothesis is also supported by the trial design, which was aimed at limiting other dietary confounding factors. Further studies are needed to confirm a direct relationship between nsLTP2 intake and hemodynamic effects in humans.

## 5. Conclusions

Based on our preliminary data, substituting modern wheat products into the diet appears to mildly but quickly improve 24-h SBP, endothelial reactivity, fasting TG, and glucose level in overall healthy subjects with suboptimal BP control. Certainly, further larger and longer-term studies are needed to confirm if the dietary intake of a bioactive peptide-rich wheat foodstuff is able to maintain the observed positive hemodynamic and metabolic effects in both prehypertensive and hypertensive subjects.

## Figures and Tables

**Table 1:** Nutrient characterization of the tested foodstuffs.

Product	Analyte	Analytical Value/100 g	
		Modern Wheat	Ancient Wheat
Wheat pasta	Energetic Value (kJ/kcal)	1446/342	1459/345
	Carbohydrates (g)	64.6	64.3
	Sugars (g)	1.2	1.1
	Protein (g)	11.7	14.8
	nsLTP2 (mg)	11.7	24.5
	Humidity (g)	11.1	11.0
	Dietary Fiber (g)	8.9	6.9
	Soluble (g)	1.8	2.1
	Insoluble (g)	7.1	4.8
	Fat (g)	2.1	1.6
	Saturated Fatty Acid (g)	0.4	0.3
	Trans Fatty Acid (g)	n.d.	n.d.
	Ash (g)	1.6	1.4
	Salt (g)	0.008	0.008
	Selenium (mg)	0.115	0.126
Matzo bread	Energetic Value (kJ/kcal)	1817/432	1798/427
	Carbohydrates (g)	67.5	66.1
	Sugars (g)	1.7	3.0
	Fat (g)	13.0	12.1
	Saturated Fatty Acid (g)	2.3	2.1
	Trans Fatty Acid (g)	n.d.	n.d.
	Protein (g)	9.6	12.1
	nsLTP2 (mg)	11.7	24.5
	Humidity (g)	4.4	4.8
	Dietary Fiber (g)	3.2	2.6
	Soluble (g)	1.2	1.2
	Insoluble (g)	2.0	1.4
	Ash (g)	2.3	2.3
	Salt (g)	1.540	1.570
	Selenium (mg)	0.055	0.079
Crisp toast	Energetic Value (kJ/kcal)	1644/390	1631/386
	Carbohydrates (g)	64.3	65.6
	Sugars (g)	3.6	3.9
	Protein (g)	13.8	13.5
	nsLTP2 (mg)	7.6	13.3
	Dietary Fiber (g)	7.6	8.0
	Soluble (g)	1.9	2.0
	Insoluble (g)	5.7	6.0
	Fat (g)	6.9	6.0
	Saturated Fatty Acid (g)	0.6	0.5
	Trans Fatty Acid (g)	n.d.	n.d.
	Humidity (g)	4.1	4.6
	Ash (g)	3.3	2.3
	Salt (g)	1.610	1.210
	Selenium (mg)	0.053	0.077

n.d.: not detected ; nsLTP2 = non-specific lipid-transfer protein type 2.

**Table 2:** Total polyphenol content and antioxidant activities of the tested ancient and modern wheats, expressed as mean ± standard deviation.

Kind of wheat	Total Polyphenol Content (mg/100 g)	2,2-Diphenyl-1-Picrylhydrazyl (DPPH) (mmol/100 g)	Fluorescence Recovery after Photobleaching (FRAP) (mmol/100 g)
Modern wheat	258.3 ± 22.3	3.9 ± 0.4	1.0 ± 0.2
Ancient wheat	230.5 ± 34.8	2.7 ± 0.8 *	1.5±0.3 *

* *p* <0.05 vs. Modern wheat.

**Table 3:** Pre-diet standardization and post-run-in parameters by assigned sequence group (modern wheat foodstuffs first or ancient wheat foodstuffs first), expressed as mean ± standard deviation.

Parameters	Pre-Diet Standardization (*N* = 63)	Post-Diet Standardization	
		Modern Wheat (*N* = 32)	Ancient Wheat (*N* = 31)
Age (years)	55.9 ± 6.8	56.3 ± 4.2	55.1 ± 6.3
Waist circumference (cm)	98.8 ± 11.3	98.2 ± 8.3	97.6±12.5
Body mass index (kg/m^2^)	27.2 ± 2.5	26.6 ± 2.2	27.1 ± 3.1
Systolic blood pressure (mmHg)	142.8 ± 6.2	136.9 ± 5.9	136.2 ± 5.6
Diastolic blood pressure (mmHg)	87.6 ± 5.1	86.4 ± 3.2	86.6 ± 3.7
Pulse pressure (mmHg)	56.2 ± 4.9	50.6 ± 2.1	49.6 ± 2.5
Mean arterial pressure (mmHg)	104.7 ± 6.8	103.2 ± 5.3	103.9 ± 5.9
Heart rate (bpm)	71.5 ± 11.2	71.7 ± 9.6	70.5 ± 10.4
Total cholesterol (mmol/L)	5.70 ± 0.88	5.46 ± 0.88	5.61 ± 0.80
Triglycerides (mmol/L)	1.46 ± 0.43	1.37 ± 0.53	1.28 ± 0.49
High-density lipoprotein cholesterol (mmol/L)	1.43 ± 0.13	1.42 ± 0.10	1.50 ± 0.09
Low-density lipoprotein cholesterol (mmol/L)	3.61 ± 0.84	3.43 ± 0.71	3.52 ± 0.79
Fasting plasma glucose (mmol/L)	4.97 ± 0.58	4.82 ± 0.55	4.82 ± 0.63
Creatinine (mcmol/L)	81.3 ± 14.4	78.7 ± 13.2	88.4 ± 10.6
eGFR (mL/s)	1.58 ± 0.12	1.55 ± 0.12	1.58 ± 0.11
Serum uric acid (mmol/L)	0.34 ± 0.08	0.32 ± 0.07	0.33 ± 0.06
Aspartate aminotransferase (mckat/L)	0.40 ± 0.09	0.34 ± 0.07	0.38 ± 0.10
Alanine aminotransferase (mckat/L)	0.44 ± 0.06	0.40 ± 0.05	0.44 ± 0.08
Urine Na^++^ (mmol/24 h)	125.5 ± 32.8	112.6 ± 21.4	119.3 ± 29.5

eGFR: Estimated glomerular filtration rate.

**Table 4:** Daily energy and nutrient intakes assessed by patient food diaries, before and after the treatment periods, expressed as mean ± standard deviation.

Variables	Modern Wheat		Ancient Wheat	
	Baseline	Post-Treatment	Baseline	Post-Treatment
Energy Value (kJ/kcal)	1483 ± 135	1492 ± 142	1470 ± 143	1514 ± 137
Carbohydrate (% of energy)	53.5 ± 3.1	54.1 ± 2.8	53.2 ± 2.9	44.2 ± 2.7
Protein (% of energy)	18.3 ± 1.9	17.1 ± 1.8	18.5 ± 1.7	16.2 ± 1.9
Animal protein (% of energy)	9.6 ± 0.8	9.3 ± 0.9	10.9 ± 0.7	9.2 ± 0.8
Vegetal protein (% of energy)	6.6 ± 0.6	5.9 ± 0.9	6.8 ± 0.8	7.3 ± 0.9
Total fat (% of energy)	28.2 ± 1.9	29.1 ± 2.1	29.4 ± 2.2	27.7 ± 2.5
Saturated fatty acids (% of energy)	9.3 ± 0.9	9.7 ± 0.6	8.9 ± 1.0	9.2 ± 1.2
MUFA (% of energy)	12.8 ± 1.1	14.6 ± 1.3	11.2 ± 1.2	13.6 ± 1.1
PUFA (% of energy)	6.5 ± 0.4	6.2 ± 0.6	6.8 ± 0.7	6.3 ± 0.4
Dietary Fiber (g)	18.1 ± 2.5	18.4 ± 1.9	17.7 ± 2.4	18.1 ± 2.6
Cholesterol (mg)	193.9 ± 14.9	189.8 ± 15.7	185.9 ± 13.7	194.2 ± 14.3

MUFA: Monounsaturated fatty acids; PUFA: Polyunsaturated fatty acids.

**Table 5:** Changes in anthropometric, hemodynamic, and hematochemistry data from baseline/end wash-out to end of treatment, expressed as mean ± standard deviation.

Parameters	Baseline/End Wash-Out		End of Treatment	
	Modern Wheat	Ancient Wheat	Modern Wheat	Ancient Wheat
Waist circumference (cm)	98.1 ± 7.8	96.4 ± 8.1	97.9 ± 7.6	95.3 ± 7.2
Body mass index (kg/m^2^)	26.5 ± 1.9	26.9 ± 2.0	26.4 ± 2.0	26.6 ± 1.8
Systolic blood pressure (mmHg)	142.3 ± 5.6	143.4 ± 5.8	141.6 ± 6.2	140.3±6.3
Diastolic blood pressure (mmHg)	85.6 ± 4.4	83.8 ± 4.7	85.1 ± 4.1	83.1 ± 3.8
Pulse pressure (mmHg)	56.1 ± 3.3	55.1±3.4	55.9 ± 3.2	56.6 ± 3.7
Mean arterial pressure (mmHg)	102.8 ± 7.0	101.4 ± 7.3	103.4 ± 6.8	100.9 ± 6.7
Heart rate (bpm)	69.6 ± 9.1	68.3 ± 8.8	70.3 ± 8.9	66.7 ± 8.1
Total cholesterol (mmol/L)	5.45 ± 0.84	5.50 ± 0.77	5.43 ± 0.87	5.44 ± 0.74
Triglycerides (mmol/L)	3.24 ± 0.89	3.08 ± 0.94	3.20 ± 0.85	2.78 ± 0.56 *^,†^
High-density lipoprotein cholesterol (mmol/L)	1.41 ± 0.09	1.49 ± 0.10	1.42 ± 0.10	1.53 ± 0.10
Low-density lipoprotein cholesterol (mmol/L)	3.40 ± 0.68	3.49 ± 0.70	3.44 ± 0.64	3.39 ± 0.69
Fasting plasma glucose (mmol/L)	2.27 ± 0.24	2.28 ± 0.23	2.24 ± 0.23	2.18 ± 0.16 *^,†^
Creatinine (mcmol/L)	77.8 ± 11.5	76.9 ± 10.6	79.6 ± 9.7	77.8 ± 10.6
eGFR (ml/s)	1.54 ± 0.12	1.56 ± 0.12	1.55 ± 0.12	1.55 ± 0.12
Serum uric acid (mmol/L)	0.14 ± 0.03	0.14 ± 0.03	0.14 ± 0.03	0.14 ± 0.03
Aspartate aminotransferase (mckat/L)	0.33 ± 0.07	0.36 ± 0.08	0.32 ± 0.08	0.34 ± 0.08
Alanine aminotransferase (mckat/L)	0.41 ± 0.06	0.42 ± 0.06	0.40 ± 0.06	0.38 ± 0.07
Urine Na^++^ (mmol/24 h)	114.8 ± 22.9	116.7 ± 24.2	113.7 ± 24.7	118.2 ± 25.9

* *p* < 0.05 vs. baseline, ^†^ *p* <0.05 vs. modern wheat foodstuffs diet; eGFR: Estimated glomerular filtration rate.

**Table 6:** Changes in 24 h ambulatory blood pressure monitoring (ABPM) parameters from baseline/end wash-out to end of treatment, expressed as mean ± standard deviation.

Parameters	Baseline/End Wash-Out		End of Treatment	
	Modern Wheat	Ancient Wheat	Modern Wheat	Ancient Wheat
SBP (mmHg)	132.9 ± 4.1	132.0 ± 5.5	131.5 ± 3.3	129.0 ± 4.1 *
DBP (mmHg)	83.4 ± 2.5	82.6 ± 3.5	84.3 ± 1.9	83.1 ± 2.7
MAP (mmHg)	100.1 ± 4.6	99.3 ± 6.6	99.5 ± 4.5	98.2 ± 4.3
PP (mmHg)	49.5 ± 4.6	49.4 ± 4.8	47.9 ± 4.1	46.9 ± 4.6
HR (bpm)	72.2 ± 6.2	72.2 ± 7.9	73.1 ± 5.5	71.0 ± 5.7
% SBP > Normal	54.3 ± 6.4	52.7 ± 6.7	54.6 ± 5.1	50.3 ± 4.3 *^,†^
% DBP > Normal	52.4 ± 6.2	49.2 ± 6.5	51.5 ± 4.9	48.4 ± 4.6
Diurnal SBP (mmHg)	137.6 ± 4.3	136.7 ± 4.7	136.3 ± 3.2	132.4 ± 3.5 *^,†^
Diurnal DBP (mmHg)	87.8 ± 5.2	87.2 ± 6.2	86.6 ± 4.5	86.1 ± 4.3
Diurnal MAP (mmHg)	104.5 ± 5.8	103.9 ± 7.2	103.6 ± 4.7	102.8 ± 5.1
Diurnal PP (mmHg)	49.9 ± 6.1	49.5 ± 5.0	48.7 ± 5.8	47.9 ± 5.4
Diurnal HR (bpm)	75.2 ± 7.1	76.0 ± 8.6	74.6 ± 5.8	74.9 ± 6.1
% Diurnal SBP > Normal	54.4 ± 6.8	52.8 ± 6.2	53.5 ± 4.7	50.2 ± 3.2 *^,†^
% Diurnal DBP > Normal	56.1 ± 6.0	54.9 ± 7.4	54.8 ± 5.3	53.9 ± 5.5
Night SBP (mmHg)	122.2 ± 4.7	120.2 ± 6.6	120.8 ± 3.4	116.4 ± 2.1 *^,†^
Night DBP (mmHg)	75.4 ± 3.3	73.3 ± 4.9	74.8 ± 3.6	71.9 ± 4.6
Night MAP (mmHg)	88.3 ± 8.2	90.2 ± 7.9	87.4 ± 5.5	88.5 ± 4.8
Night PP (mmHg)	49.2 ± 4.9	49.2 ± 5.6	48.3 ± 3.5	47.8 ± 4.4
Night HR (ppm)	65.6 ± 6.4	64.5 ± 7.5	64.4 ± 4.8	62.8 ± 4.6
% Night SBP > Normal	46.5 ± 6.3	42.5 ± 7.4	45.8 ± 4.6	40.6 ± 4.1 *^,†^
% Night DBP > Normal	36.4 ± 6.4	32.3 ± 7.2	35.7 ± 4.9	31.8 ± 5.3

* *p *< 0.05 vs. baseline, ^†^ *p* <0.05 vs. modern wheat foodstuffs diet; DBP: Diastolic blood pressure; HR: Heart rate; MAP: Mean arterial pressure; PP: Pulse pressure; SBP: Systolic blood pressure.

**Table 7:** Changes in endothelial function and arterial stiffness parameters from baseline/end washout to end of treatment, expressed as mean ± standard deviation.

Parameters	Baseline/End Wash-Out		End of Treatment	
	Modern Wheat	Ancient Wheat	Modern Wheat	Ancient Wheat
Pulse wave velocity (m/s)	9.5 ± 1.9	9.7 ± 2.1	9.6 ± 2.0	9.5 ± 1.3
Augmentation index (%)	24.5 ± 2.6	26.0 ± 3.5	25.7 ± 2.7	25.6 ± 3.1
Pulse volume changes (%)	65.4 ± 6.1	63.9 ± 6.3	64.3 ± 6.6	68.1 ± 4.2 *^,^^†^

* *p* <0.05 vs. baseline, ^†^ *p* <0.05 vs. modern wheat foodstuffs diet.
